# Knowledge and Asset Management in Sustainable Civil Engineering

**DOI:** 10.1155/2014/307970

**Published:** 2014-04-14

**Authors:** Eddie W. L. Cheng, Neal Ryan, Yat Hung Chiang

**Affiliations:** ^1^Department of Social Sciences, The Hong Kong Institute of Education, 10 Lo Ping Road, Tai Po, Hong Kong; ^2^Curtin University Sustainability Policy Institute, Curtin University, Bentley, Perth, WA 6102, Australia; ^3^Department of Building and Real Estate, The Hong Kong Polytechnic University, Hunghom, Kowloon, Hong Kong

This special issue aims at fostering the dissemination of high-quality research in methods, theories, and techniques concerning the application of knowledge and asset management in sustainable civil engineering. Similar to that of other special issues, the selection process was rigorous and only articles with good quality could be accepted. We, as editors, would like to thank all of those who had submitted their articles for possible publication. Some articles with good quality were not chosen because they were not definitely relevant to the issue scopes. Some others that were relevant were excluded because some modification work was required. Unfortunately, the stringent reviewing guideline set for the special issue puts us in a situation that we could not accept articles with major revision. Thus, we had to sacrifice these articles although they had the potential to be published in the special issue. Finally, six articles were published in this special issue, which brings together contributions from authors originating from various geographical locations including Australia, China, Korea, Sweden, and Taiwan.

This special issue has published four research and two review articles. The article by Y. Liu et al. discusses the use of case-based reasoning and variable fuzzy sets to develop appropriate safe early warning systems for highway construction projects in China. They argue that there is a lack of study of production safe early warning, especially early warning in construction projects. They realize that good early warning technologies can help reduce losses by restricting the possible accident sources and can indirectly lower investment costs by maximizing the benefit from the safety inputs. In their article, they introduce the key technology for safe early warning systems and explain the concepts of association rules, support vector machine, and variable fuzzy qualitative and quantitative change criterion mode. They provide solutions for solving three key issues of index selection, accident cause association analysis, and warning degree forecast, through the above concepts, to improve the highway construction early safe warning systems.

The other three articles are related to the application of building information modeling (BIM) for civil engineering projects. Y.-C. Lin and Y.-C. Su studied facility maintenance management (FMM) for the operation phase of the construction life cycle in Taiwan. They envisage the use of BIM to support the maintenance service of facilities depicted in 3D object-oriented CAD. Specifically, the BIM approach is used to store facility information in a digital format, which can facilitate easy updates of FMM information in a 3D CAD environment. They introduce the novel BIM-based FMM (BIMFMM) system for the acquisition and tracking of maintenance information. This system acts as a platform that enables maintenance staff to share information through webcam-enabled notebook or tablet. They demonstrate a case study, which was to apply the system in a commercial building in Taiwan, to evaluate the effectiveness of the system. Their combined results support that the system is a useful BIM-based FMM platform that integrates web and BIM technologies for handling FMM.

S.-P. Ho et al., a research group from National Taiwan University, also propose the application of building information modeling (BIM) technology to share construction knowledge. They introduce a BIM-based knowledge sharing management (BIMKSM) system. In this knowledge-based sharing system, construction knowledge can be communicated and reused among project managers and jobsite engineers. The BIM technology further supports the generation of 3D drawings that are particularly useful for identifying knowledge and experience feedback relevant to construction projects. The authors expect that this system helps alleviate problems on a construction jobsite and reduces the time and cost of solving problems related to constructability. In their article, the system was applied in a case study of a construction project in Taiwan to demonstrate the sharing of knowledge within the BIM environment.

Another article by R. A. Kivits and C. Furneaux focuses on the important concept of building information management (BIM), which as they quote is “the use of virtual building information models to develop building design solutions, to design documentation, and to analyze construction processes.” Their paper outlines the current promise and future potential for BIM and makes recommendations in relation to how the problems can be addressed. They also argue that the concept can be applied to other civil infrastructure projects, such as dams, bridges, and tunnels, despite its primary link to building projects. A set of cases are presented to indicate how BIM has been implemented. Their paper further suggests that BIM has the potential for improving all stages of the construction life cycle and has implications for both sustainability and asset management.

The study by S. Lee et al. is intended to develop a financing model to solve financial barriers for implementing green building projects in the Republic of Korea. They highlight the importance of green buildings that reduce greenhouse gas energy but realize the difficulty in attracting private funding. Their financing model takes the governmental support into account by incorporating the government guarantee for the increased costs of a green building project in return for certified emission reduction (CER). One of the main components in the model is to compare between the value of the governmental guarantee and that of CER This can make sure that the government can secure return from its guarantee. An example has been demonstrated to show how the payback period can be calculated using the model. Such an asset management approach can help finance sustainable green building projects. Further, the authors argue that private guarantees may also be feasible given the promising value of the guarantee from CER.

The last, but not least valued, study by A. Warsame et al. draws the attention to the role of knowledge management and incentives that can improve the quality of transport infrastructure projects. They underscore the role played by a client organization on developing a knowledge culture for dealing with procurement and organizational issues. They argue that all procurement methods can give good results if the right conditions are given. They then introduce the knowledge conversion modes and identify the four major conditions from the knowledge management perspective. They also discuss the use of incentives to motivate employees to generate the behavior appropriate for knowledge management. They further propose that the client organization should seek independent second opinions from external actors in a systematic way during all stages of an infrastructure project in order to improve knowledge and create incentives.

Now, when our work, as guest editors, comes to an end, we hand in to our readers this special issue that was bred on several seeds, in which we firmly believe that they bring to the world new insights, ideas, and recommendations. We finally hope that readers will enjoy reading the special issue, dedicated to exploring knowledge and asset management in sustainable civil engineering.

*Eddie W. L. Cheng* *Eddie W. L. Cheng* *Neal Ryan* *Neal Ryan* *Yat Hung Chiang* *Yat Hung Chiang*

